# Tuning and Coupling Irreversible Electroosmotic Water Flow in Ionic Diodes: Methylation of an Intrinsically Microporous Polyamine (PIM-EA-TB)

**DOI:** 10.1021/acsami.3c10220

**Published:** 2023-08-28

**Authors:** Zhongkai Li, John P. Lowe, Philip J. Fletcher, Mariolino Carta, Neil B. McKeown, Frank Marken

**Affiliations:** † Department of Chemistry, University of Bath, Claverton Down, Bath BA2 7AY, U.K.; ‡ Materials & Chemistry Characterisation Facility, MC^2^, University of Bath, Bath BA2 7AY, U.K.; § Department of Chemistry, Swansea University, College of Science, Grove Building, Singleton Park, Swansea SA2 8PP, U.K.; ∥ EaStCHEM School of Chemistry, University of Edinburgh, Joseph Black Building, David Brewster Road, Edinburgh, Scotland EH9 3JF, U.K.

**Keywords:** electroosmosis, ionomer membranes, coupled diodes, rectification, voltammetry

## Abstract

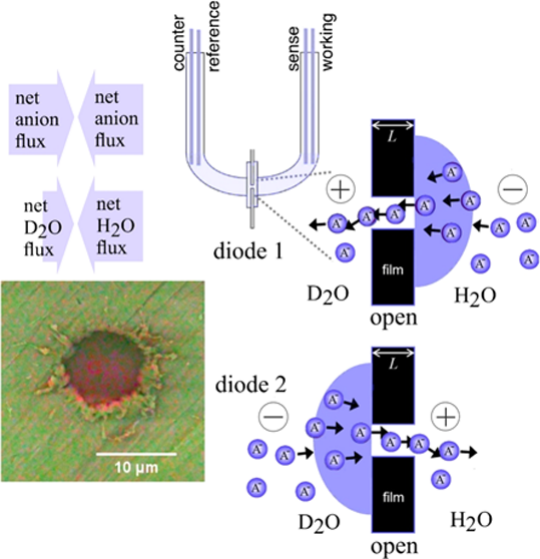

Molecularly rigid polymers with internal charges (positive charges induced by amine methylation) allow electroosmotic water flow to be tuned by adjusting the charge density (the degree of methylation). Here, a microporous polyamine (PIM-EA-TB) is methylated to give a molecularly rigid anion conductor. The electroosmotic drag coefficient (the number of water molecules transported per anion) is shown to increase with a lower degree of methylation. Net water transport (without charge flow) in a coupled anionic diode circuit is demonstrated based on combining low and high electroosmotic drag coefficient materials. The AC-electricity-driven net process offers water transport (or transport of other neutral species, e.g., drugs) with net zero ion transport and without driver electrode side reactions.

## 1 Introduction

Electroosmotic phenomena are caused by ion transport being associated with water transport.^1^ This is sometimes undesirable (e.g., in fuel cells^2^) and sometimes desirable (e.g., in electroosmotic pumps for drug delivery^3^). The electroosmotic transport of water has been studied in considerable depth^4,5^ due to water being extremely important technically, for example, in decontamination technologies.^6^ Electroosmotic transport of water during direct current (DC) electrodialysis^7^ is always associated with electrolysis at driver electrodes, which causes unwanted side products and energy losses as well as potential degradation/damage to drug delivery devices. An alternative methodology based on alternating current (AC) electricity could be useful, but it requires a net zero ion movement and irreversibility (i.e., diode-like behavior) in the transport. Here, it is shown that coupling two ionic diodes^8^ with differing electroosmotic drag coefficients into an ionic circuit offers a new way to realize net water transport driven by electricity.

**Figure 1:**
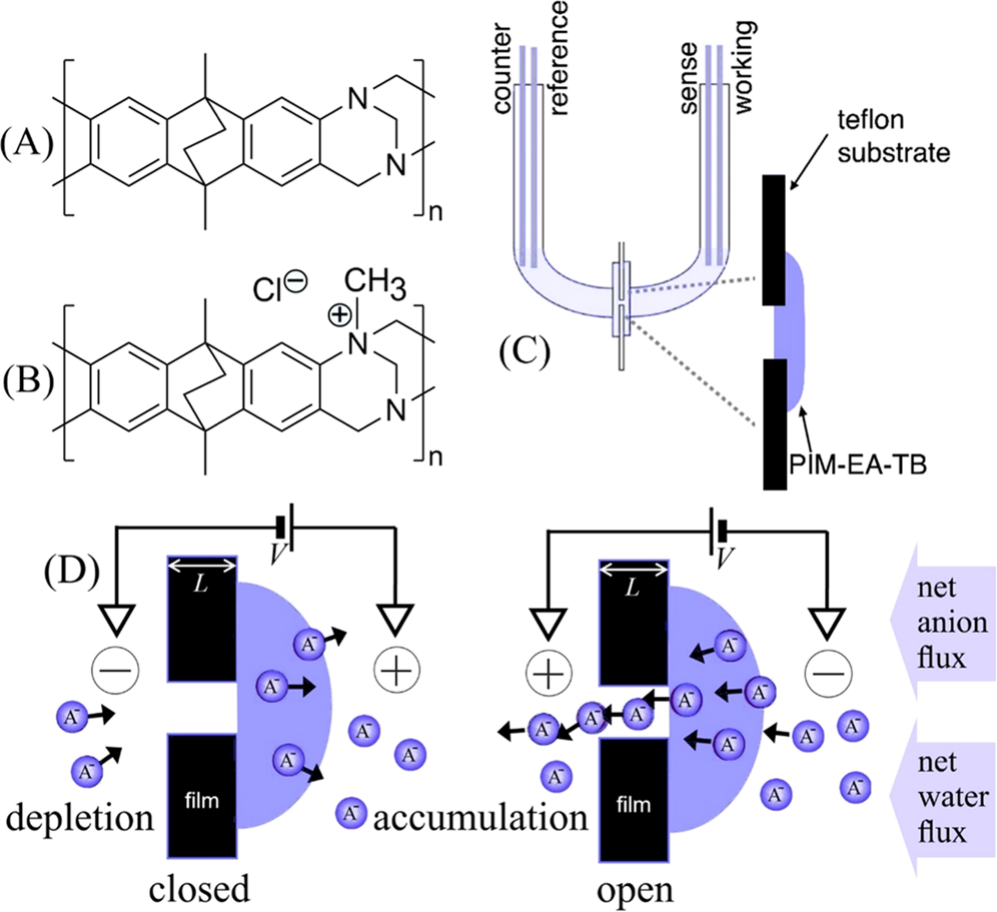
(A) Molecular structure of PIM-EA-TB. (B) Molecular structure of methylated PIM-EA-TB. (C) 4-Electrode ionic diode experimental configuration. (D) Schematic of switching of anionic diodes between closed and open states.

Polymers of intrinsic microporosity offer an innovative class of molecularly rigid polymer materials with interesting membrane properties.^9,10^ In a previous study of electrochemical properties, protonation of the polymer of intrinsic microporosity PIM-EA-TB (see the molecular structure in Figure 1A) was shown to lead to both anionic diode behavior and coupled to this substantial electroosmotic water transport.^11^ The electroosmotic drag coefficient (the number of water molecules transported per anion) was shown to be strongly pH-dependent and suggested to increase with a lower degree of protonation (i.e., a higher charge density in the rigid polymer lowers the electroosmotic drag coefficient). This leads to the realization that molecular rigidity in the PIM-EA-TB framework is responsible for anions and water moving concertedly. Anions behave similar to “mechanical pistons” that move the water through the rigid host framework. However, for many applications, the pH dependence of this water transport process is undesirable (e.g., the degree of protonation can change locally in pores during operation with externally applied potentials). Therefore, methylation of the amine site in PIM-EA-TB could provide an alternative more chemically robust approach to tuning the charge density independent from solution pH. This should also allow tuning of the electroosmotic water transport.

Figure 1B shows the molecular structure of the methylated PIM-EA-TB. Methylation is readily achieved, for example, with iodomethane or other alkylation reagents.^12,13^ Once methylated, the intrinsically microporous polymer becomes anion conducting. Figure 1C shows the experimental configuration for ionic diode measurements. The PIM-EA-TB is placed onto an inert substrate material (here, 5 μm thick Teflon) with a laser-drilled microhole (typically 10 μm diameter). An externally applied potential will induce anion transport either from PIM-EA-TB into the microhole (electrolyte accumulation leads to an “open” diode) or out of the microhole into PIM-EA-TB (electrolyte depletion leads to a “closed” diode; Figure 1D).^14^ Here, a microhole-based ionic diode is employed, but there are other types of ionic diodes reported (based on nanopores,^15^ nanocones,^16^ or microfluidic devices^17^) with similar functionality.

The net result of anionic diode operation is a unidirectional/irreversible flux of anions. Each anion transfer is associated with the transfer of water molecules. For ionic diodes to be useful in devices, it is necessary to couple them into pairs/circuits to employ both positive and negative polarization periods during processes driven by AC electricity. It was shown recently that the combination of an anionic diode with a cationic diode offers an approach to net salination/desalination processes.^18^ Similarly, here it is demonstrated that coupling two anionic diodes based on PIM-EA-TB with a differing degree of methylation results in a process of net zero flux of anions coupled to a net unidirectional flux of water. This type of AC-electricity-energized water pumping process can be considered biomimetic^19^ and potentially useful for water purification or water recovery/drying processes or beneficial in a range of drug delivery or water harvesting applications.^20^

## 2 Experimental Section

### 2.1 Chemical Reagents

The PIM-EA-TB polymer (Sigma-Aldrich 918784; molecular weight approximately 70 KD; monomer weight for C_21_H_20_N_2_ 300 g mol^–1^; density approximately 1.1–1.3 g cm^–3^ or 3.7–4.3 mmol cm^–3^;^21^ pore volume typically 30–26% (FFV),^22^ therefore wet density approximately 1.4–1.7 g cm^–3^) was synthesized following a previously reported method.^23^ Iodomethane (99%), NaClO_4_ (≥98.0%), D_2_O (99.9% atom D, contains 0.05 wt % 3-(trimethylsilyl)propionic-2,2,3,3-d_4_ acid, sodium salt as the internal standard), dimethyl sulfoxide, DMSO-*d*_6_ (99.9 atom % D), and chloroform were purchased from Sigma-Aldrich. NaCl (99.5%) was purchased from Fisher Scientific Ltd. Methanol (HPLC) was purchased from VWR Chemicals BDH. Agarose powder was purchased from Melford Ltd. All reagents were applied and used as received without further purification. All aqueous solutions were prepared with ultrapure water with a resistivity not less than 18.2 MΩ·cm (20 °C), from a CE Instruments water purification system.

### 2.2 Instrumentation

**Figure 2:**
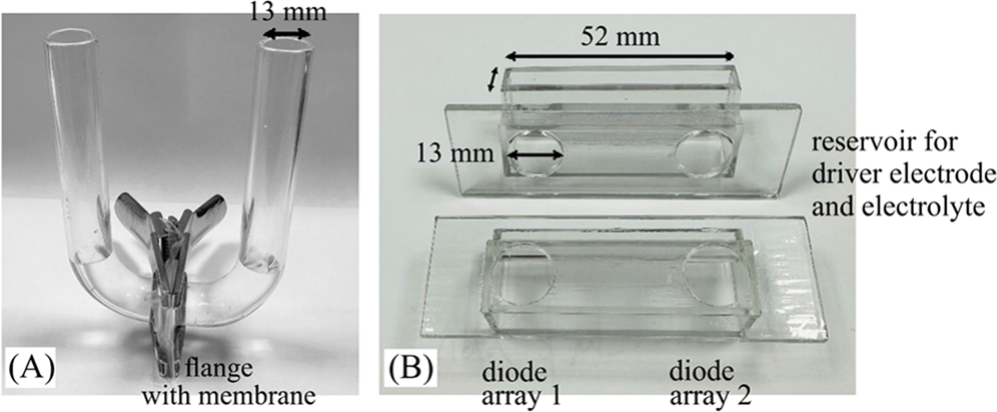
Photograph of (A) a U-cell with flange and membrane and (B) a 3D-printed (transparent resin) device cell for two coupled arrays of anionic diodes.

Electrochemical characterizations including cyclic voltammetry, chronoamperometry, and impedance spectrometry were carried out on a computer-controlled Ivium Technologies CompactStat.h potentiostat, with a classic four-electrode configuration. Electroosmotic transport of water in both single and coupled diode systems were performed with an Autolab PGSTAT12 potentiostat (Metrohm). Single-microhole experiments were performed with a U-cell (Figure 2A). Microhole array experiments were performed either in a U-cell for single ionic diode investigations or with a 3D-printed transparent resin cell (i.materialise.com; Figure 2B) for a coupled ionic diode system. Membrane electrochemical cells consisted of two half-cells or reservoirs separated by a 5 μm thick Teflon film (Laser Machining Ltd, U.K.), which was laser-drilled with either a single microhole (approximately 10 μm diameter) or an array of 200 microholes (two arrays of 10 × 10 microholes, pitch 200 μm). Carbon rods (1 mm diameter) were applied as both working and counter electrodes, and silver wires (0.5 mm diameter) served as both quasi-sense and quasi-reference electrodes. During all measurements, the working and sense electrodes were placed on the membrane side of the cell. Scanning electron microscopy (SEM) and energy-dispersive X-ray spectroscopy (EDX) of the membrane-coated microhole were performed on a Hitachi SU3900 variable pressure SEM with an attached Oxford Instruments Ultim Max 170 mm^2^ EDX detector. The detection of H_2_O in D_2_O was performed by ^1^H NMR spectrometry and D_2_O in H_2_O by ^2^H NMR (Bruker 400 MHz).

### 2.3 Procedures

#### 2.3.1 Membrane Preparation

A chloroform solution of 3 mg mL^–1^ PIM-EA-TB was prepared, from which 10 μL was deposited onto the microhole region of the Teflon film to form a single microhole. A volume of 40 μL was used (coated over roughly 4 times the area) for an array of microholes. Before film deposition, the Teflon film was placed on a glass substrate of 1 wt % agarose gel to prevent the PIM-EA-TB solution from penetrating into or through the microhole. After evaporation, a polymer membrane was formed as an asymmetric coating over the microhole. Consistent with previous experiments,^24^ the typical thickness was 10 ± 5 μm (based on cross-sectional SEM data).

#### 2.3.2 PIM-EA-TB Methylation

The methylation of PIM-EA-TB followed a method similar to that reported for another TB-based polymer.^25^ A Teflon film with a PIM-EA-TB membrane was transferred onto a watch glass, where a methanolic solution of iodomethane (typically 0.1 M, but in some cases, the concentration was varied) was used to cover the polymer membrane. The watch glass was then sealed in a bigger glass dish (with the bottom covered with methanol to saturate the surrounding air and to minimize the evaporative losses of the iodomethane solution). The reaction was kept in dark for 20 h (at room temperature), before the container was reopened and the membrane recovered and rinsed with methanol (warning: *all steps have to be performed in a fumehood environment and with personal protection due to iodomethane being highly toxic*). After drying in air, the membrane was exposed to aqueous 10 mM NaCl to equilibrate and to exchange the iodide against chloride anions. For electrochemical measurements, the Teflon film with the polymer membrane was placed between two reservoirs to construct the electrochemical cell. For single-microhole experiments, 0.01 M of aqueous electrolyte (NaCl or NaClO_4_) was placed symmetrically in both reservoirs of the cell (Figure 2).

#### 2.3.3 Energy-Dispersive X-ray (EDX) Spectral Analysis

Single-microhole membrane samples were prepared with PIM-EA-TB or with methylated PIM-EA-TB (using 1 M iodomethane in methanol). The membranes were immersed in 0.01 M aqueous NaCl for 1 h to allow ion exchange/equilibration before rinsing, drying, and submission for scanning electron microscopy (SEM) imaging and EDX elemental mapping. Similarly, PIM-EA-TB membranes (on a silicon wafer) were prepared with different levels of methylation. These were also equilibrated in aqueous 0.01 M NaCl prior to SEM/EDX measurements.

#### 2.3.4 Electroosmotic Drag Coefficient Measurements

A previously reported^11^ approach based on nuclear magnetic resonance (^1^H NMR) was applied to monitor the electroosmotic flow (potential driven or without potential bias) through the PIM-EA-TB membrane. A Teflon film with an array of 200 microholes (each approximately 10 μm diameter) was used to increase the currents/fluxes. An asymmetric coating of methylated PIM-EA-TB was prepared in the same way as described for the single-microhole system. A volume of 10 mL of aqueous 0.01 M electrolyte (NaCl or NaClO_4_) was added into the working electrode chamber, and a volume of 10 mL of 0.01 M electrolyte in D_2_O (with DMSO as the internal standard) was added into the counter electrode chamber. This allowed the transport of H_2_O to be monitored with/without applied voltage.

During measurements, the cell was first allowed to stand for 90 min without external voltage. During this time the diffusional transport of H_2_O into D_2_O was monitored (background). Then, a fixed voltage of −1 V (corresponding to the open state of the anionic diode with high current) was applied across the membrane for 150 min. Both diffusional and electroosmotic transport occurred and were monitored. Finally, the cell was allowed to rest for 90 min without applied potential. The diffusional transport was monitored. Samples of 0.6 mL of D_2_O solution were taken from the cell with 30 min intervals. Good mixing was ensured before sampling. The samples were then submitted for ^1^H NMR measurement to analyze the concentration of H_2_O in D_2_O, by integration of peaks at 2.65 ppm for dimethyl sulfoxide (DMSO) and at 4.70 ppm for protons in H_2_O/D_2_O. The electroosmotic drag coefficient was determined by combining the ^1^H NMR and electrochemical charge data.

#### 2.3.5 AC-Electroosmotic Pumping of Water

Two PIM-EA-TB/Teflon diodes (200 microholes each) with different levels of methylation were prepared by using different concentrations of methanolic iodomethane, one with 1 M solution (for low water flux) and the other with 0.01 M solution (for high water flux). The Teflon films were then placed in between two 3D-printed half-cells (Figure 2B) facing in opposite directions. A volume of 10 mL of 0.01 M NaCl in H_2_O (with DMSO-*d*_6_ as the internal standard) was added to the working electrode chamber, and a volume of 10 mL of 0.01 M NaCl in D_2_O (with DMSO as the internal standard) was added to the counter electrode chamber. During measurement, first, the system was allowed to stand for 90 min without current applied to monitor the diffusional transport (background). Then, an AC signal was applied to the system to achieve net zero ion transport through two anionic diodes with unidirectional water transport. A driving current of ± 24 μA was employed in the form of square wave pulses (10 s duration) to enforce symmetric charge transport. The current was applied over a period of 120 min. Samples of 0.6 mL solution were taken from both chambers with 30 min intervals. Good mixing was ensured prior to taking samples. D_2_O solution samples were then submitted for ^1^H NMR measurement to analyze the concentration of H_2_O in D_2_O and H_2_O solution samples for ^2^H NMR to analyze the concentration of D_2_O in H_2_O.

## 3 Results and Discussion

### 3.1 Effect of PIM-EA-TB Methylation on Ion Transport: Single-Microhole Experiments

**Figure 3:**
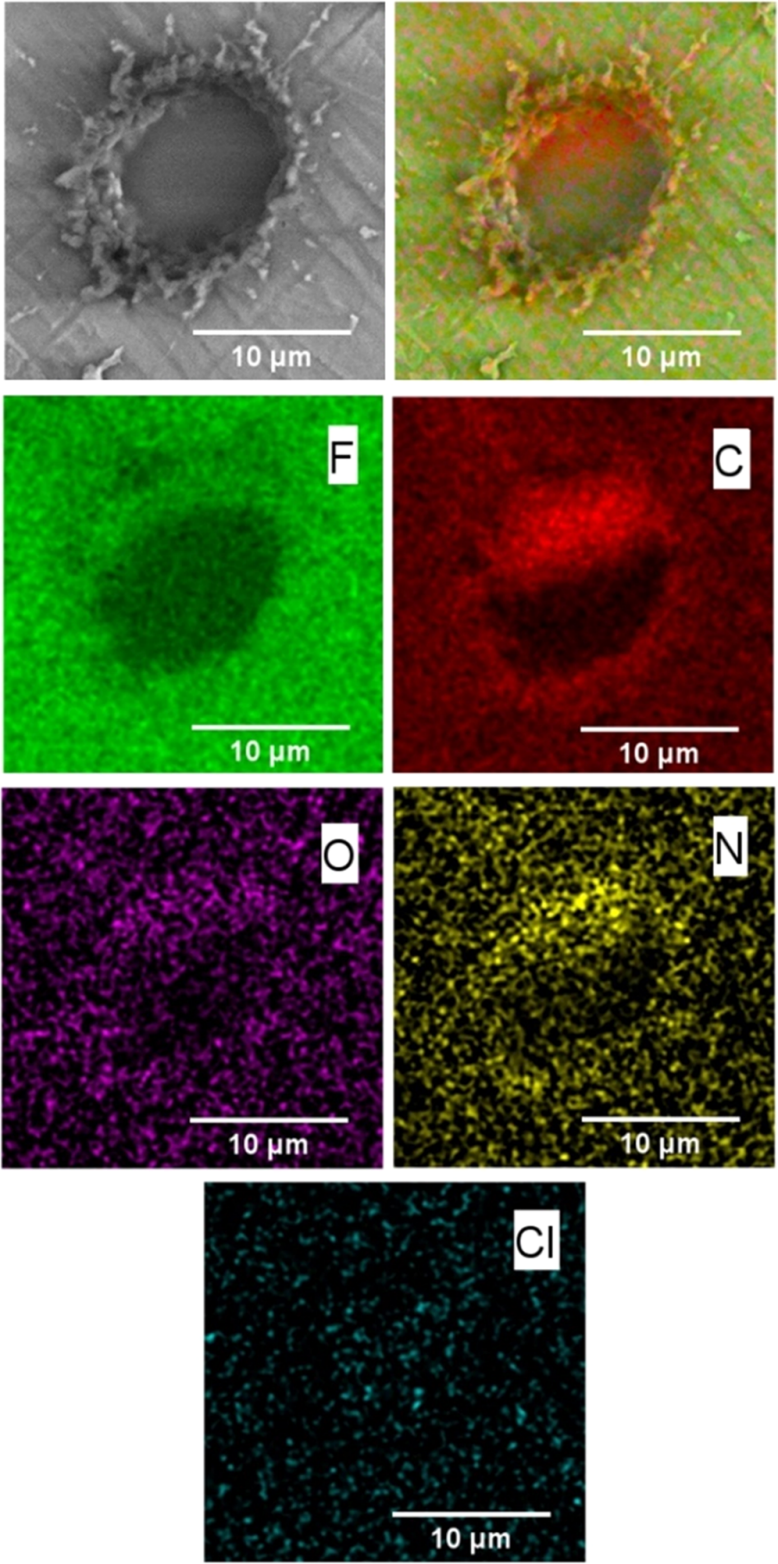
Scanning electron microscopy (SEM, backscatter) images of the backside of a Teflon film with 10 μm microhole coated with a methylated PIM-EA-TB (with 0.1 M iodomethane) membrane. EDX elemental mapping for F, C, O, N, and Cl (shadowing due to the detector position).

The microporous polymer PIM-EA-TB is applied to the inert substrate (5 μm thick Teflon) prior to the methylation step. Once methylation is performed (employing 0.1 M iodomethane in methanol; 20 h), the polymer becomes insoluble and nonprocessable. Figure 3 shows a typical scanning electron micrograph (SEM) for PIM-EA-TB after methylation (shown through the Teflon substrate microhole). Energy-dispersive X-ray (EDX) elemental analysis and mapping show prevalence of fluorine on the Teflon and prevalence of carbon on the PIM-EA-TB. The presence of nitrogen and the presence of chloride are indicative of PIM-EA-TB being present and of successful methylation (the iodide from the reaction with iodomethane was exchanged against chloride by immersion into aqueous 10 mM NaCl). However, the quality of the elemental mapping is limited (see Table 1) and therefore additional more quantitative EDX experiments were performed with PIM-EA-TB films on a substrate without microholes (Table 2).

**Table 1:** EDX Mapping Elemental Data for Microhole Samples Coated with PIM-EA-TB with/without Methylation (0.1 M Iodomethane in Methanol; 20 h)

	microhole EDX mapping atomic %						
sample	C	O	N	Cl	F (Teflon)	Cu (substrate)	total
PIM-EA-TB	31.5	1.0	0.5	0.0	63.9	3.1	100.0
methylated PIM-EA-TB	38.1	0.8	0.6	0.03	58.9	1.6	100.0

**Table 2:** EDX Mapping Elemental Data for Teflon Film Samples Coated with PIM-EA-TB with/without Methylation (Various Concentrations of Iodomethane in Methanol; 20 h)

	membrane deposition EDX atomic %						atomic ratio	
meI conc./mM	C	O	N	Cl	I	Cl + I	Cl/N	(Cl + I)/N
0	83.1	1.27	6.14	0.00	0.00	0.00	0.00	0.00
1	88.0	1.48	7.04	0.09	0.00	0.09	0.013	0.013
10	88.1	2.07	7.63	0.70	0.31	1.01	0.092	0.132
25	87.6	1.78	8.07	1.54	0.44	1.98	0.191	0.245
50	85.8	1.94	7.41	2.00	0.59	2.59	0.270	0.349
100	84.7	2.12	6.45	2.00	1.11	3.11	0.310	0.482

**Figure 4:**
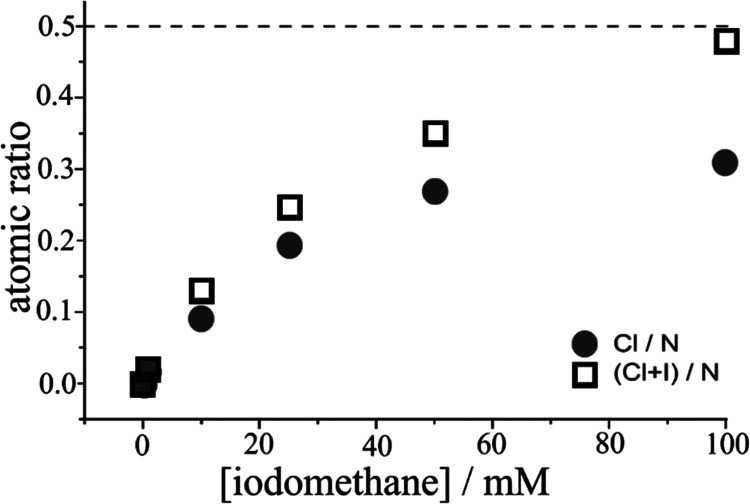
Plot of EDX elemental data for PIM-EA-TB films methylated with different concentrations of iodomethane immersed in 0.01 M aqueous NaCl and ions exchanged for 1 h.

When changing the concentration of the methylation reagent (iodomethane), the degree of methylation in the process can be controlled. Data in Table 2 demonstrate that a concentration of 0.1 M iodomethane results in essentially complete methylation (one methyl group per PIM-EA-TB monomer unit). The ion exchange from iodide to chloride was not exhaustive, and therefore, both Cl and I are detected in the sample. The atomic ratio parameter (Cl + I)/N is employed to demonstrate methylation of up to 50% of the nitrogen atoms in the molecular structure. Each PIM-EA-TB monomer contains two nitrogen atoms, but only one of these is methylated (see Figure 1B). Figure 4 shows a plot with the degree of methylation reaching approximately half of the monomer units for 25 mM iodomethane reagent. The degree of methylation converges toward full conversion for one nitrogen atom in every monomer unit for 0.1 M iodomethane. It is therefore possible to prepare ionomer films with both, a low or a high degree of methylation to compare their properties.

**Figure 5:**
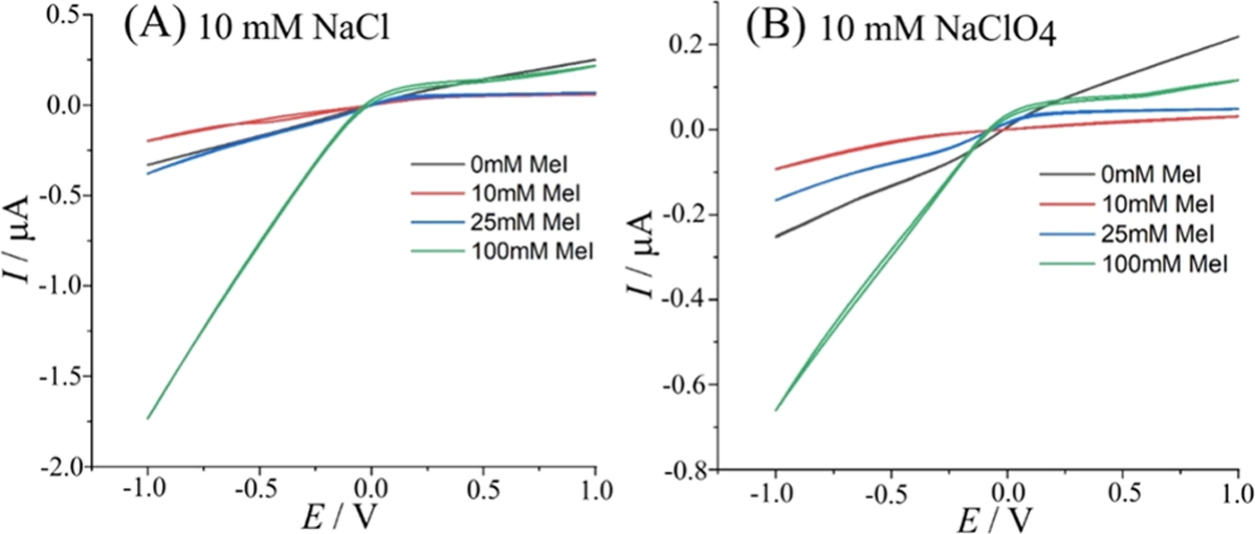
(A) Cyclic voltammetry (scan rate 200 mV s^–1^) data for PIM-EA-TB immersed in 10 mM NaCl as a function of degree of methylation. (B) As before but immersed in 10 mM NaClO_4_.

The effect of methylation of PIM-EA-TB is clearly observed in 4-electrode cyclic voltammetry experiments. Figure 5 shows data for PIM-EA-TB (thickness typically 10 ± 5 μm) on a microhole (10 μm diameter) on a Teflon substrate (5 μm thickness). Immersed in aqueous 10 mM NaCl and without methylation, there is no significant diode effect (Figure 5A). An essentially Ohmic characteristic is observed due to resistivity in the microhole region. With 10 and 25 mM iodomethane treatments, the diode effect gradually emerges and improves. Asymmetry in the current response is observed with significant current in the negative potential region (diode open) and low current in the positive potential region (diode closed). With 100 mM iodomethane treatment, a clear anionic diode is observed with a rectification ratio of 8.0 at ± 1 V bias. Increasing the degree of methylation significantly increases the current (for both open and closed diode) due to the higher anion conductivity of the ionomer.

A similar set of 4-electrode cyclic voltammograms was recorded in aqueous 10 mM NaClO_4_ (Figure 5B) to explore potential effects from anion interactions with the methylated PIM-EA-TB. Currents in 10 mM NaClO_4_ are generally lower consistent with a slower migration of anions through the methylated PIM-EA-TB (lower conductivity due to stronger ClO_4_^–^ anion interactions with the partially hydrophobic polymer host^26^). However, the effect of methylation on diode character is similar with a rectification ratio of typically 5.7 at ± 1 V for a 100 mM iodomethane-treated PIM-EA-TB film.

**Figure 6:**
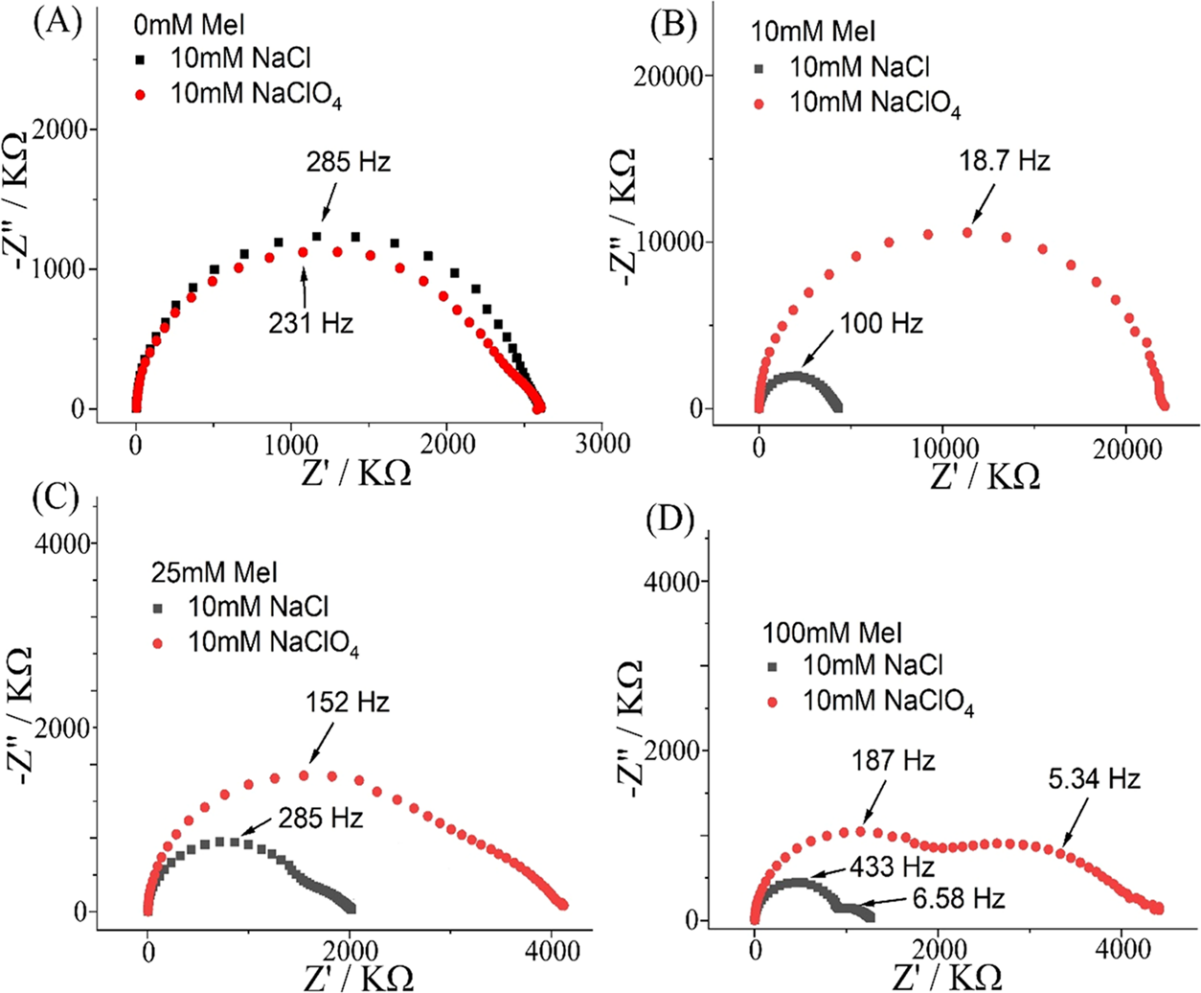
Data from electrochemical impedance spectroscopy (20 mV amplitude; 0.0 V bias) comparing data in 10 mM NaCl (black) and data in 10 mM NaClO_4_ (red). (A) No methylation. (B) Methylation with 10 mM iodomethane. (C) Methylation with 25 mM iodomethane. (D) Methylation with 100 mM iodomethane. Indicated are high/low-frequency summit frequencies.

Electrochemical impedance spectroscopy can provide further insight into the ionic diode performance. Figure 6 shows electrochemical impedance spectroscopy data (Nyquist plots) comparing frequency responses in aqueous 10 mM NaCl and in aqueous 10 mM NaClO_4_ for different degrees of PIM-EA-TB methylation. Typical ionic diode frequency responses are based on two semicircular features: one semicircle at higher frequencies and one semicircle at lower frequencies. Both, the high- and low-frequency semicircles are associated with a “summit frequency” *f*_diode_ and a corresponding time constant τ_diode_ = *R* × *C* (Figure 6 and eq 1).
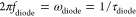
1Physically, the high-frequency semicircle is linked to the charging of the Teflon substrate film (typical capacitance *C* = 0.3 nF) with discharge via the microhole (resistance *R*_2_ ranging from 2.5 to 0.9 MΩ; Table 3). An increase in the degree of methylation lowers the resistivity of the microporous ionomer, and thereby it lowers the high-frequency time constant (Figure 6).

**Table 3:** Electrochemical Impedance Spectroscopy (EIS) Data Fitting Parameters Including Semicircle Summit Frequencies and Time Constants

	(A) 10 mM NaCl				
level of meI (mM)	*R*_1_ (Ω)	*R*_2_ (Ω)	*C*_1_ (F)	freq 1 (Hz)	freq 2 (Hz)
0	4.36 × 10^3^	2.52 × 10^6^	2.38 × 10^–10^	285^a^	
10	4.62 × 10^2^	3.96 × 10^6^	3.68 × 10^–10^	100	
25	4.03 × 10^3^	1.55 × 10^6^	3.08 × 10^–10^	285	
100	4.17 × 10^3^	9.08 × 10^5^	3.30 × 10^–10^	433	6.6

	(B) 10 mM NaClO_4_				
level of meI (mM)	*R*_1_ (Ω)	*R*_2_ (Ω)	*C*_1_ (F)	freq 1 (Hz)	freq 2 (Hz)
0	5.29 × 10^3^	2.32 × 10^6^	2.53 × 10^–10^	231^a^	
10	5.05 × 10^3^	2.17 × 10^7^	3.67 × 10^–10^	18.7	
25	4.34 × 10^3^	3.06 × 10^6^	3.17 × 10^–10^	152	
100	4.14 × 10^3^	2.11 × 10^6^	3.29 × 10^–10^	187	5.3

a Values can be affected/elevated by uncontrolled protonation in the absence of methylation.

The low-frequency semicircular feature can be attributed to the switching of the ionic diode between open and closed states. Without methylation or with low levels of methylation (10 mM iodomethane), the diode switching effect is not observed (visible as a secondary low-frequency semicircle in the Nyquist plot). For 100 mM iodomethane treatment, the two semicircles are both clearly observed. The second semicircular feature reflects the diode switching process. This can be represented by the time constant τ_diode_ or the summit frequency *f*_diode_ (Figure 6). Values for 10 mM NaCl (*f*_diode_ = 6.6 Hz; τ_diode_ = 24 ms) and for 10 mM NaClO_4_ (*f*_diode_ = 5.3 Hz; τ_diode_ = 30 ms) are very similar and consistent with previous observations made with 10 μm diameter microhole diodes.^27^ The switching time constant is dominated by diffusion–migration of the electrolyte into/out of the microhole region and less sensitive to the ionomer properties.

### 3.2 Effect of PIM-EA-TB Methylation on Ion Transport: Microhole Arrays

**Figure 7:**
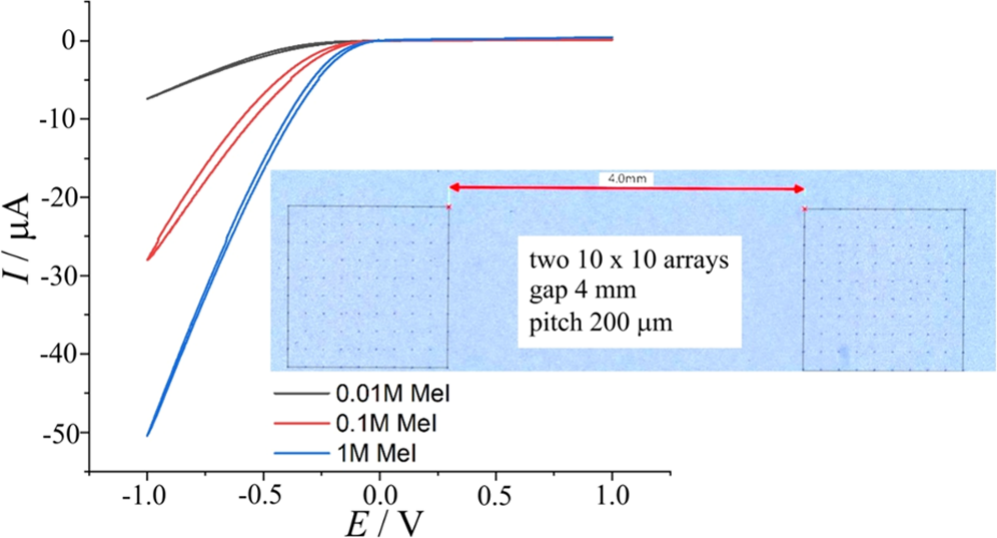
Cyclic voltammograms (scan rate 200 mV s^–1^) for PIM-EA-TB diodes (for three levels of methylation) immersed in aqueous 10 mM NaCl.

Next, experiments are performed with double arrays of 100 microholes each (each separated by 200 μm pitch to limit the diffusional overlap). Figure 7 shows a photograph of the two arrays separated by a 4 mm gap. Figure 7 also shows cyclic voltammetry data for 200 microhole arrays coated with PIM-EA-TB as a function of degree of methylation (immersed in 10 mM NaCl). Diode currents increase with the degree of methylation.

When compared to data obtained at a single microhole (Figure 5A), the array currents are higher typically by a factor 20 (but not by a factor 200 probably due to some diffusional overlap). The degree of methylation clearly improves the diode performance with 1 M iodomethane producing a well-defined anionic diode with a rectification ratio of 108 at ± 1 V bias. For this type of array of 200 diodes, it is now possible to follow the transport of water and to determine (at least in the first approximation) the electroosmotic drag coefficients associated with the different degrees of methylation.

### 3.3 Effect of PIM-EA-TB Methylation on Electroosmotic Water Transport: Microhole Arrays

**Figure 8:**
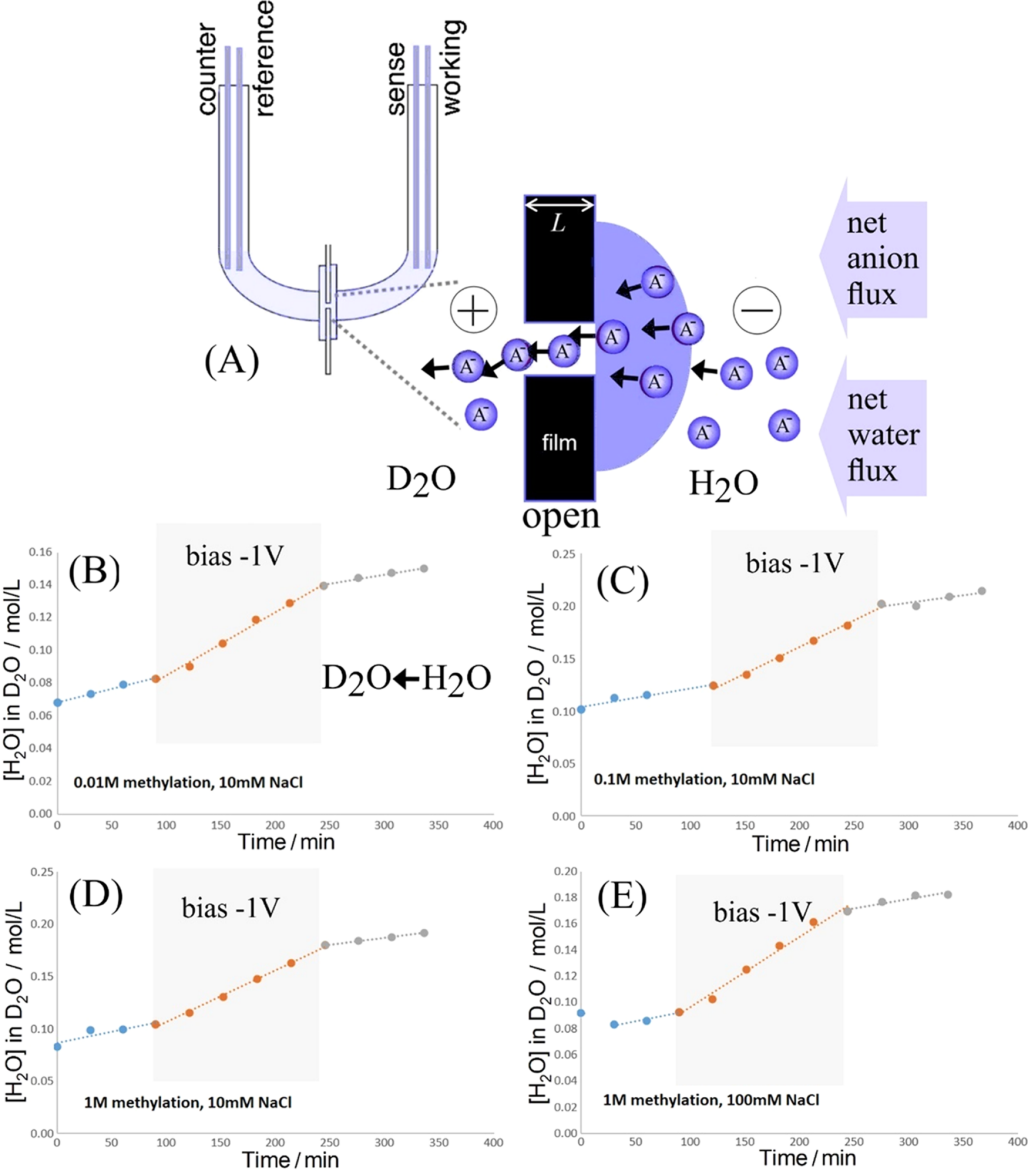
(A) Illustration of H_2_O transport (into D_2_O) together with anion transport through the open diode with −1 V applied bias (for an array of 200 microholes). (B) PIM-EA-TB methylation with 0.01 M iodomethane; transport in 10 mM NaCl. (C) PIM-EA-TB methylation with 0.1 M iodomethane; transport in 10 mM NaCl. (D) PIM-EA-TB methylation with 1 M iodomethane; transport in 10 mM NaCl. (E) PIM-EA-TB methylation with 1 M iodomethane; transport in 0.1 M NaCl (−1 V applied bias indicated in gray).

Anion transport is associated with the simultaneous transport of water molecules (either associated with the anion or “pushed along”). The corresponding transport of water for the “open” diode or for the “closed” diode is assumed to be proportional to the current and therefore linked to the rectification ratio. Rectified ion transport is associated with rectified water transport. In order to determine the electroosmotic drag coefficient (the number of water molecules transported per anion), the measurement cell is filled with H_2_O solution on one side and with D_2_O on the opposite side. The transfer of anions can be monitored by the flow of current, and the transfer of water molecules can be monitored by nuclear magnetic resonance (NMR) spectroscopy. PIM-EA-TB was coated onto arrays of 200 microholes, treated with iodomethane, treated with 10 mM NaCl to exchange anions, and then employed in water transport measurements (Figure 8).

There is a substantial diffusional flux of H_2_O across the membrane without applied bias voltage (which includes the uptake of atmospheric H_2_O; *vide infra*). This appears to be relatively unchanged upon methylation. The background diffusional flux of H_2_O is mainly entropy-driven across the membrane with approximately 10 μm thickness. An estimate of the Fickian membrane diffusion coefficient *D*_H_2_O_ = 2 ± 1 × 10^–9^ m^2^s^–1^ (this value seems high probably due to ignoring the uptake of atmospheric water; very similar to previous estimates^10^) can be obtained based on eq 2.
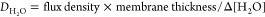
2With the bias voltage of −1 V applied, the current through the open diode is significant (Table 4) and the water flux increases. For the closed diode, the current is much lower, and additional water flux is therefore insignificant (relative to the background diffusional flux of water). The electroosmotic flux was obtained by subtracting the background diffusional water flux from the experimentally observed flux. Data in Table 4 summarize how the current increases with the degree of methylation. Perhaps surprisingly, an increase in methylation leads to a higher current but also to a lower electroosmotic drag coefficient. This trend is very similar to that observed for protonated PIM-EA-TB.^11^ Also, the magnitude of the electroosmotic drag coefficient comparing protonated and methylated PIM-EA-TB seems similar or slightly lower for the methylated materials when compared to the protonated material. The mechanism suggested for explaining the electroosmotic drag coefficient as a function of charge density in PIM-EA-TB was linked to the molecularly rigid (glassy) nature of the polymer. In the absence of polymer chain movements, the anions have to “push” the water through the pores. For a fully methylated PIM-EA-TB, the charge density, 4000 mol m^–3^, can be estimated from the molecular weight of the monomer unit, 300 g mol^–1^, and the density of the PIM-EA-TB polymer (assuming little effect of the methylation). The accessible pore volume in PIM-EA-TB suggests 22,000 mol m^–3^ water. The ratio suggests 5 water molecules per charge for fully methylated materials. However, swelling in the aqueous environment will lead to a considerable increase in this value (Table 4). A lower degree of methylation should further increase the electroosmotic drag coefficient.

**Table 4:** Data Summary for H_2_O Transport Experiments Based on Monitoring H_2_O Flux into D_2_O over 150 min (See Figure 8)

meI conc. (M)	electrolyte	average *I*/*Q*	electroosmosis of H_2_O with anions (mmol)	H_2_O transported per anion
0.01	10 mM NaCl	6.31 μA/54.9 mC	0.35	640
0.1	10 mM NaCl	21.3 μA/192 mC	0.51	278
1	10 mM NaCl	37.5 μA/337 mC	0.45	138
1	100 mM NaCl	536 μA/4820 mC	0.54	72
1	10 mM NaClO_4_	28.8 μA/258 mC	0.30	119

Included in Table 4 is a data set for the electroosmotic water transport comparing aqueous 10 mM NaCl and aqueous 10 mM NaClO_4_ environments. Although the current in the presence of perchlorate is somewhat lower, the electroosmotic drag coefficient appears effectively unchanged. This confirms that the charge density and the pore volume are the key parameters to explain the electroosmotic drag coefficient. The nature of the anion affects the current but not the water transport per anion.

### 3.4 AC-Electroosmotic Pumping of Water: Coupling Two Microhole Arrays

It has recently been demonstrated that ionic diodes can be coupled into ionic circuits to perform AC (alternating current)-electricity-driven functions. When combining an anionic diode with a cationic diode, a net transport of salt (anions and cations) is achieved in ionic diode desalination processes.^18^ Similarly, it is possible to couple two anionic diodes with different electroosmotic drag coefficients to perform a water pumping function (with net zero ion flow).^11^ This type of process could be of interest in water recovery (compare water recovery mechanisms in biological systems^19^) or in drug dosing or supply.^28^

**Figure 9:**
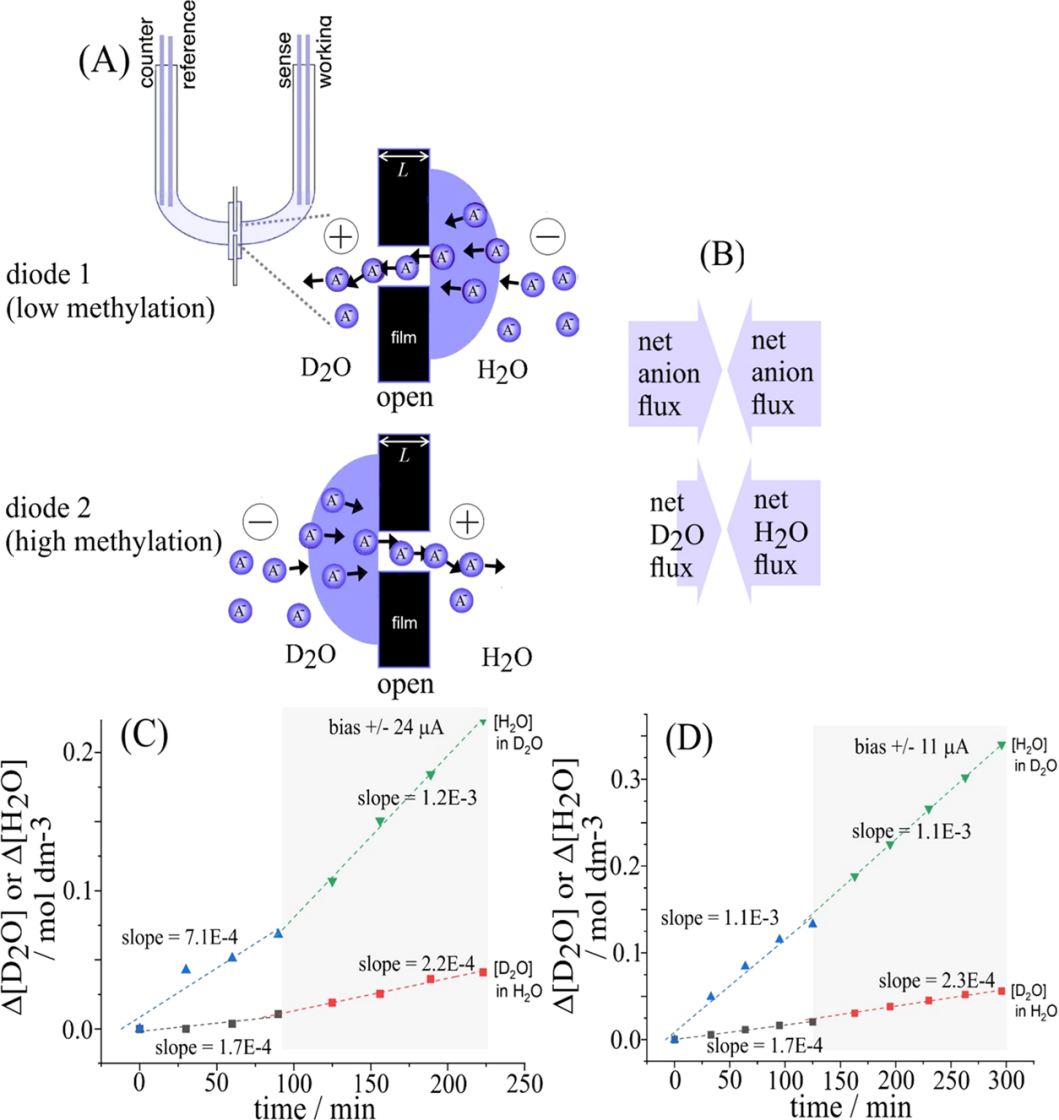
(A) Illustration of two coupled anionic diodes operating in opposite directions. When diode 1 is open, then diode 2 is closed. The charges flowing through the two diodes are fixed by chronoamperometry. The associated flow of water will be higher in diode 1 (low degree of methylation) and lower in diode 2 (higher degree of methylation). (B) Diagram to summarize the effect of net water flow for net zero charge transport. (C) Experimental data for 10 mM NaCl in H_2_O (working electrode compartment; 10 mL) and 10 mM NaCl in D_2_O (counter electrode compartment; 10 mL). The current was set to ± 24 μA with a 0.1 Hz switching rate. (D) Experimental data for 10 mM NaCl in D_2_O (working electrode compartment; 10 mL) and 10 mM NaCl in H_2_O (counter electrode compartment; 10 mL). The current was set to ± 11 μA with a 0.1 Hz switching rate.

Here, we use the effect of the degree of methylation to couple two anionic diodes with low/high electroosmotic water drag coefficients. Figure 9A illustrates this process. A fixed current is applied to the driver electrodes for a fixed period of time (polarity switching every 10 s) to define the charge passing through the coupled diodes. The 3D-printed cell shown in Figure 2B is employed with arrays of 200 microholes for each diode linking the two compartments.

Data in Figure 9C suggest that water transport through the PIM-EA-TB with lower charge density (methylation with 0.01 M iodomethane) is higher compared to the water transport through the PIM-EA-TB with a higher degree of methylation (1 M iodomethane). Both, the transport of H_2_O into D_2_O and the transport of D_2_O into H_2_O were monitored (the background for H_2_O seems higher probably due to the uptake of atmospheric H_2_O; further minor isotope effects on water transport are possible, but they are ignored here; *vide infra*). Given the electroosmotic drag coefficients determined for these types of membranes, the predicted rate of water pumping (coupling PIM-EA-TB methylated in 1 M iodomethane and PIM-EA-TB methylated in 0.01 M iodomethane) would be 640 – 138 = 502 water molecules per ion cycle (see Table 4; one ion cycle = one anion going to the right and one anion going to the left).

The difference in slope in Figure 9C multiplied with the volume and Faraday constant and divided by the current gives the observed electroosmotic drag coefficient. The observed water transport (for 24 μA applied current; selected to keep the driving voltage below ± 1.2 V) for the [H_2_O] transport is 308 and for the [D_2_O] transport is 80. The difference 308 – 80 = 228 water molecules per ion cycle represents the excess water transport and therefore the net water pumping effect (given equal anion flow between compartments). This suggests 50% efficiency, given the values for electroosmotic drag coefficients determined above. The data suggest the proof of principle for water transport in coupled anionic diodes. It is interesting to invert the experiment and to replace H_2_O with D_2_O and *vice versa*. Data in Figure 9D show that now the transport of H_2_O through highly methylated PIM-EA-TB is low (within error of the experiment), whereas the transport of D_2_O through less-methylated PIM-EA-TB is slightly increased (at much lower currents). Overall, the results agree with each other (suggesting only very minor isotope effects). For future improvements in water transport efficiency, higher currents will be desirable.

## 4 Conclusions

It has been shown that anionic diodes are produced by methylation of PIM-EA-TB. The degree of methylation changes the density of charge in the microporous polymer and thereby allows the electroosmotic drag coefficient to be tuned. A lower degree of methylation increases the electroosmotic drag and water transport. In contrast, a higher degree of methylation decreases the electroosmotic drag coefficient. As a result of this, coupling of two anionic diodes into an ionic circuit results in a net zero flow of anions with a substantial net flow of water. By selecting ionic diodes with high and low degrees of methylation, the net water transport is considerable and potentially useful in applications such as water extraction, water purification, or dosing of drugs in aqueous solutions. In essence, the process can be compared to reverse osmosis but driven without the need to apply external pressure.
